# Predicting brain age using Tri-UNet and various MRI scale features

**DOI:** 10.1038/s41598-024-63998-6

**Published:** 2024-06-14

**Authors:** Yu Pang, Yihuai Cai, Zonghui Xia, Xujie Gao

**Affiliations:** 1 School of Science, Jilin Institute of Chemical Technology, Jilin, 130000 China; 2 Faculty of Artificial Intelligence in Education, Central China Normal University, Wuhan, 430079 China; 3 School of Information Science and Technology, Beijing Forestry University, Beijing, 100083 China

**Keywords:** Computational biology and bioinformatics, Diseases, Health care, Engineering

## Abstract

In the process of human aging, significant age-related changes occur in brain tissue. To assist individuals in assessing the degree of brain aging, screening for disease risks, and further diagnosing age-related diseases, it is crucial to develop an accurate method for predicting brain age. This paper proposes a multi-scale feature fusion method called Tri-UNet based on the U-Net network structure, as well as a brain region information fusion method based on multi-channel input networks. These methods address the issue of insufficient image feature learning in brain neuroimaging data. They can effectively utilize features at different scales of MRI and fully leverage feature information from different regions of the brain. In the end, experiments were conducted on the Cam-CAN dataset, resulting in a minimum Mean Absolute Error (MAE) of 7.46. The results demonstrate that this method provides a new approach to feature learning at different scales in brain age prediction tasks, contributing to the advancement of the field and holding significance for practical applications in the context of elderly education.

## Introduction

The structure of the human brain undergoes gradual changes throughout the entire lifespan^1^. Knickmeyer et al.^2^, in a longitudinal study of brain structure MRI in healthy individuals, found that the brain volume of newborns is typically only half that of adults, increasing to 90% of adult size by adulthood. Research by Good et al.^3^ indicates a gradual decrease in gray matter volume throughout adulthood, with white matter volume following an inverted “U-shaped” curve, reaching its peak in middle age.

Binte et al.^4^ conducted a morphological analysis of brain structure using structural MRI data from 619 participants aged 19.98 to 83.62 years in the publicly available IXI dataset. The study revealed that as humans reach middle age, the brain undergoes aging with increasing age, accompanied by structural atrophy. The most significant manifestation of this aging process is a decline in cognitive abilities. Furthermore, brain aging increases the risk of brain diseases, especially age-related neurological disorders such as mild cognitive impairment, Alzheimer's disease, schizophrenia, epilepsy, traumatic brain injury^5–10^.

The various diseases that accompany brain aging impose significant burdens on individuals, families, and society as a whole. While aging is inevitable, interventions in early life can be employed to prevent or reduce brain damage, potentially preventing common neurological diseases^11–13^. Therefore, accurately assessing the degree of individual brain aging to effectively identify the brain's health status has become a crucial research topic.

With the rapid development of deep learning technology and its expanding applications, an increasing number of researchers are employing deep learning methods to predict brain age. Jiang et al.^14^ obtained brain MRI data from a total of 1454 healthy subjects aged 18–90 from five publicly available datasets. The data were split into a training set with 1303 samples and a test set with 151 samples. The researchers then segmented the brain into seven different functional networks using the cortical parcellation template (CorticalParcellation_Yeo2011). Subsequently, three methods—3D CNN, Gaussian Process Regression (GPR), and Relevance Vector Regression (RVR)—were employed to train models. All three methods performed best on the Frontoparietal Network (FPN), Dorsal Attention Network (DAN), and Default Mode Network (DMN) brain networks. The 3D CNN method demonstrated average Mean Absolute Errors (MAE) of 5.55 years, 5.77 years, and 6.07 years for the three networks, outperforming the machine learning methods GPR and RVR. This work suggests that Convolutional Neural Networks (CNNs) hold significant potential for predicting brain age.

Bintsi et al.^15^ proposed a patch-based brain age prediction framework using an ensemble approach of 3D Convolutional Neural Network (CNN) and linear regression. The model was trained on the UK Biobank dataset, yielding a final average MAE of 2.46 years. By utilizing patches, the researchers identified brain regions with the greatest impact on brain age prediction, providing interpretable results and advancing anatomical research in the context of deep learning for brain age prediction. This study confirmed the hippocampus and regions such as the ventricles as most relevant to age prediction.

Traditional brain age prediction methods based on anatomical measurements overlook the biological spatial information of brain anatomical structures. This results in a challenging spatial information gap in machine learning-based brain age prediction methods, even for leading algorithms that exhibit significant measurement errors. Because these methods lack context information from neighboring voxels and are insensitive to nonlinear relationships^16^, researchers are increasingly turning their attention to Convolutional Neural Networks (CNNs) in the field of deep learning^17^.

Ballester et al.^18^ proposed a multi-channel network model based on ResNet18. They used segmented gray and white matter slices as input data for two channels, constructing a convolutional neural network. The final brain age prediction was obtained by averaging the results of three independently trained neural networks. Despite improvements in the MAE evaluation metric in this study, the use of 2D slices as input data prevents the network from integrating contextual information from different brain regions. Additionally, the selection of slices is subject to human factors, leading to significant changes in prediction accuracy when different slice indices are chosen, resulting in a lack of stable predictive performance. Pardakhti et al.^19^, using a 3D ResNet, achieved the best MAE of approximately 5 years. However, this method only provides whole-brain age predictions, whereas current research on brain age prediction is moving towards regional visualization, requiring voxel-level predictions for assessing regional changes in brain aging and lesion locations related to age-related diseases^20,21^.

Therefore, Popescu et al.^22^, based on the U-Net network for image segmentation followed by voxel-level brain age prediction, achieved the best MAE of around 7 years in the frontal cortex and periventricular regions. However, this method solely employs U-Net for training without effectively integrating regional information between different feature layers. Their utilization of multi-scale features is not thorough, and identical features in different brain regions may represent different information, leading to the omission of many features and the loss of substantial spatial information.

To address these issues, this paper proposes a 3D network architecture, Tri-UNet, based on 3D U-Net and 3D ResNet. Brain region data is utilized as input features for the multi-input network to make it more suitable for brain age prediction tasks. The effectiveness of the proposed method is validated on the publicly available Cam-CAN dataset, demonstrating good accuracy.

The main contributions of this paper are as follows:

1. Proposed a brain age prediction method based on the 3D U-Net network structure and residual learning, addressing the issue of insufficient feature utilization in predicting brain age using the U-Net network. This method enhances the utilization of features by repeatedly reusing features through residual connections from ResNet on the foundation of U-Net. It strengthens the correlation between features at different scales, achieving maximal feature utilization and demonstrating good performance.
2. Proposed a brain age prediction method based on a multi-channel input network, addressing the issue of information redundancy in previous studies using multi-channel input networks to predict brain age. In this paper, the original 3D brain imaging data were segmented into anatomical regions defined by experts, and the segmented brain regions were used as input data for the network. This allows the network to learn specific parameters for different brain regions, capturing region-specific atrophy patterns. Consequently, it integrates complementary information from different brain regions, improving the accuracy of brain age prediction while reducing training time.

**Figure 1:**
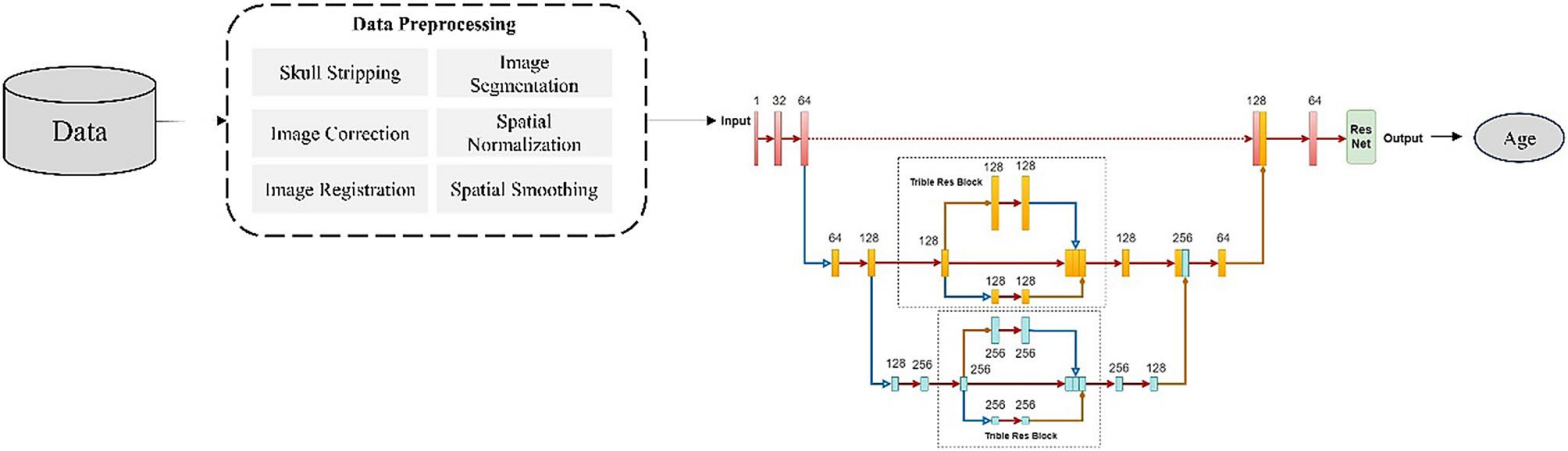
Experimental flowchart.

The paper is organized as follow: The Materials and Methods section describes the dataset, data preprocessing techniques, the Tri-UNet model and the Multi-channel input network model. The Result and Discussion section extensively elaborated on the achievements of the Tri-UNet model in both the whole brain and specific brain regions, accompanied by in-depth analysis of its outcomes. Additionally, the effectiveness of using the whole brain and individual brain regions as inputs to the multi-channel model was explored and validated using the ADNI dataset. Lastly, correlations between different brain regions and the degree of brain aging were investigated. Figure 1 shows the flowchart of our experiment.

## Materials and methods

### Dataset

The neuroimaging data utilized in this investigation emanate from the publicly accessible Cambridge Centre for Ageing and Neuroscience (Cam-CAN) data repository. This paper utilized T1-weighted MRI data from 651 healthy participants (male/female = 322/329, mean age = 54.7 ± 18.6, age range 18–89 years) from the Cam-CAN repository, as shown in Table 1.

**Table 1:** Description of Cam-CAN.

Dataset	Samples	Age	Average age	Agender (Men/Women)
Cam-CAN	651	18.5–88.9	54.7 ± 18.6	322/329

**Figure 2:**
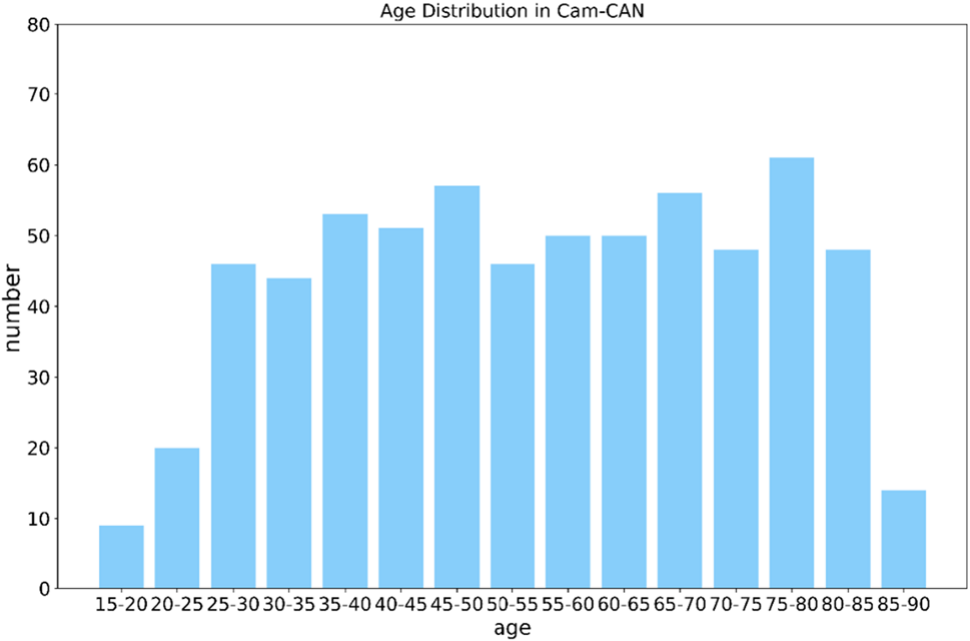
Age distribution of Cam-CAN participants.

To examine the age distribution of participants in the Cam-CAN dataset, the HC (Cam-CAN) data was categorized into groups of 5 years each, resulting in the age distribution graph shown in Fig. 2. All MRI data were acquired from a 3 T Siemens TIM Trio scanner equipped with a 32-channel head coil. The scanner utilized a 3D magnetization-prepared rapid gradient-echo (MPRAGE) sequence to obtain high-resolution 3D T1-weighted images, with the following parameters: TR = 2250 ms, TE = 2.99 ms, inversion time TI = 900 ms, flip angle FA = 9°; field of view FOV = 256 × 240 × 192 mm^3^, slices = 192; voxel size = 1 mm^3^ isotropic; GRAPPA acceleration factor = 2; scan time TA = 4 min 32 s.

The data for this project adhered to exclusion criteria similar to other datasets, excluding individuals with neurological disorders (e.g., Parkinson's disease, Alzheimer's disease), psychiatric disorders (e.g., depression), hematologic disorders (e.g., anemia, leukemia), or a history of traumatic brain injury. Additionally, participants with conditions such as migraines, diabetes, or tinnitus were excluded, and only healthy individuals meeting these criteria were included. Ethical approval was obtained locally for data collection in the Cam-CAN project, and subsequently, an anonymized version of the data was made publicly available.

The Alzheimer's Disease Neuroimaging Initiative (ADNI) is a longitudinal multicenter research project aimed at developing biomarkers for early detection and tracking of Alzheimer's disease (AD). The project has recruited participants from across North America (at least 50 regions in the United States and Canada) who consented to undergo various imaging and clinical assessments. ADNI aims to assist researchers and clinicians in diagnosing the progression of AD by measuring the sensitivity and specificity of early biomarkers, facilitating the development of new therapeutic approaches, monitoring treatment efficacy, and improving the efficiency and safety of drug development, thereby reducing the time and cost of clinical trials.

In this study, researchers used T1-weighted MRI data for the task of predicting brain age and categorized participants into three groups: Healthy Controls (HC), Mild Cognitive Impairment (MCI), and Alzheimer's Disease (AD).

### Data preprocessing

**Figure 3:**
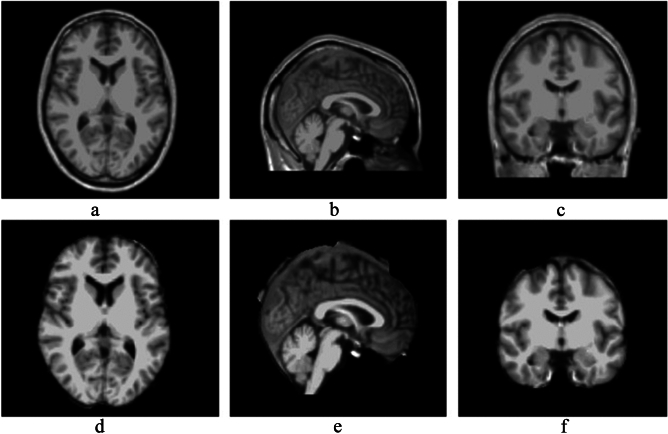
(**a**–**c**) The original image. (**e**–**g**) The result of preprocessing.

Typically, in tasks involving predicting brain age based on neuroimaging data, the original images undergo preprocessing steps before being input into the network^23^. This involves slicing the original 3D images into 2D images for network input. However, this approach loses contextual information in the feature space, leading to lower prediction accuracy compared to directly inputting 3D images. Therefore, this study exclusively employs the fully automated processing pipeline "recon-all" from the medical image processing software Freesurfer for the preprocessing of original images. This includes steps such as skull stripping, image correction, image registration, image segmentation, spatial normalization, and spatial smoothing, as illustrated in Fig. 3, which contrasts the preprocessed image with the original image.

### U-Net

U-Net is a convolutional neural network model initially introduced by Ronneberger et al.^24^ in 2015 for image segmentation. Its structure is symmetrical, resembling the uppercase letter “U,” and consists of encoding and decoding stages with skip connections in between. The encoding stage performs downsampling, while the decoding stage performs upsampling. The number of feature channels doubles in the encoding stage and halves in the decoding stage. The U-Net's multi-level encoding–decoding structure can combine deep and shallow features, enabling better learning of contextual semantic information from input features. This not only enhances prediction accuracy but also allows the model to learn fine-grained features.

U-Net has found widespread application in the field of image segmentation^25^. However, since medical imaging data is often in three-dimensional format, U-Net needs to slice the 3D data before training, leading to the loss of spatial contextual features in medical images.

Therefore, Çiçek et al.^26^ proposed 3D U-Net, a network structure similar to 2D U-Net but designed for three-dimensional data. In this study, the architecture of 3D U-Net is adopted to construct the framework for brain age prediction.

In addition, U-Net still has some shortcomings. UNet networks typically rely on pixel-level feature extraction for image processing, higher-level abstract features are required for brain age prediction. Considering the relatively shallow structure of UNet networks, they may struggle to effectively handle complex brain image data, potentially leading to capacity limitations.

### Tri-UNet

Currently, in the work utilizing the U-Net network for predicting brain age, there is a lack of sufficient utilization of features at different scales. For intricate brain structures and age-related features, UNet networks may not offer sufficient model capacity to capture these characteristics. UNet primarily depends on local features and skip connections for image segmentation, whereas brain age prediction necessitates more global and comprehensive features. Consequently, UNet networks may excessively rely on local features, neglecting the broader contextual information within the images, thus resulting in insufficient adaptability for brain age prediction tasks.

**Figure 4:**
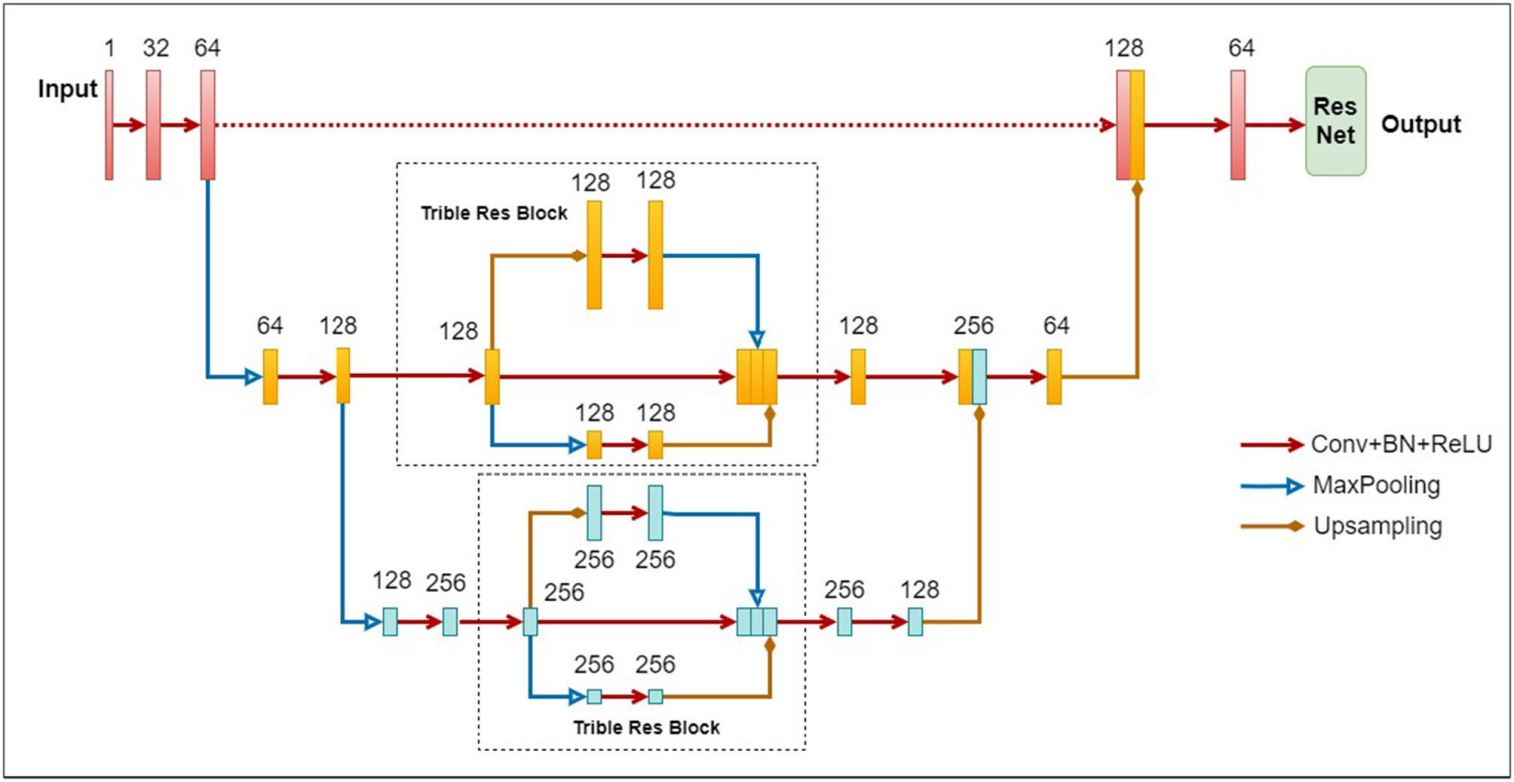
The architecture of Tri-UNet.

Therefore, in order to address the aforementioned shortcomings, this paper proposes a network model named Tri-UNet, based on 3D ResNet and 3D U-Net, for the task of brain age prediction. By incorporating ideas from Inception and skip connections, the paper proposes a Three-Branch Residual Block (Trible Res Block). The Trible Res Block allows each feature layer to capture information from both higher and lower layers, addressing the issue of degradation in deep networks. Finally, the tail of Tri-UNet is appended with a ResNet 34 network, utilized for predicting brain age based on the features learned by the encoding–decoding structure of Tri-UNet. The network model structure is illustrated in Fig. 4.

### Trible Res Block

As indicated by the two dashed boxes in Fig. 4, the model structure of the Trible Res Block includes three paths. The first path is the normal input path, which performs no operations, preserving the content of the input features. The second path is the upsampling path, which enlarges the size of the input features. It then undergoes two convolutional block operations with the aim of learning large-sized features after information restoration, thereby enhancing information fitting capabilities. The third path is the downsampling path, which reduces the size of the input feature map through max-pooling. It then executes two basic block operations to learn deep features.

After the three paths undergo convolutional operations, the resulting feature maps are concatenated together. It is important to note that the upsampling path needs to undergo downsampling to reduce the size of the feature map before concatenation, while the downsampling path requires upsampling to increase the size of the feature map. This ensures alignment of the feature map sizes from the three paths before concatenation. Finally, the concatenated feature map set is used as the output.

Following the Trible Res Block operation, the original input features carry both deep and shallow information simultaneously. The Trible Res Block merges the original input features with the upsampling and downsampling features, enhancing the correlation between upper and lower-level information.

### Multi-channel input network model

#### Model structure

**Figure 5:**
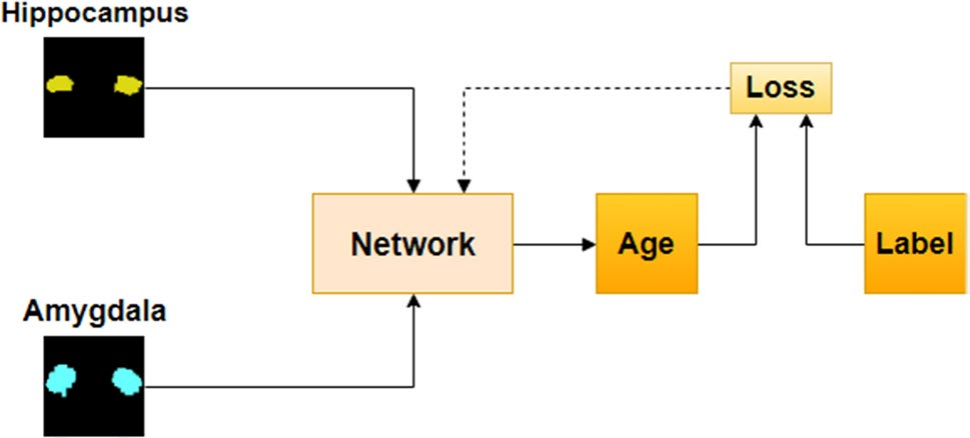
Multi-channel input network architecture diagram.

In the multi-channel input network model, the network can learn regional features from several brain regions and use them to regressively predict brain age. Because different brain regions can provide additional complementary information^27^, the neural network in the multi-channel input network model can capture more regional information, thereby improving the accuracy of brain age prediction. Firstly, based on biological prior knowledge, this study identified the brain regions most correlated with brain age, the hippocampus and amygdala. These brain structures were chosen because they are highly sensitive to physiological stress and known to be affected by pathological processes related to cognitive aging and dementia, thus serving as good indicators of subtle individual differences in health and biology^28^. Meanwhile, studies have shown significant differences in the hippocampus and amygdala among AD, MCI, and HC, and indicate that these differences increase with age. Additionally, the divergence between control and pathological models of these structures emerged as early as around 40–45 years old, with the distance between models gradually increasing relative to age^29^. Then, multiple-channel inputs are fed into the network, consisting of MRI segmentation maps of brain regions that have a significant impact on the accuracy of brain age prediction. Finally, the convolutional neural network produces the ultimate predicted age. The network model is illustrated in Fig. 5. Here, "Network" can be any convolutional neural network, and in this paper, a 3D ResNet34 network model is employed.

### Brain region segmentation

Many studies indicate that during the process of brain aging, the extent of atrophy varies in different brain regions^30,31^. Therefore, instead of using the entire brain, this paper utilizes several brain regions most relevant to predicting brain age as input data for the prediction framework to obtain regional estimates of brain age. The network can focus more on specific brain regions without being influenced by other parts of the brain. Personalized brain regions can exhibit more sensitive changes with the development of diseases or in response to treatment effects, providing a more detailed representation of changes in brain structure over time^32^. This allows deep learning network models to achieve interpretable results and serves as a reference for more localized brain age predictions. Finally, by combining features from different brain regions, the multi-channel input network model produces more robust predictions of brain age.

### Experimental design

To validate the effectiveness of the two proposed methods in this paper, comparative experiments were conducted. First, to verify the effectiveness of the improvements on the U-Net network model, whole-brain cropped images were used as input data for the Tri-UNet model to obtain predictions of brain age. These predictions were then compared with the method proposed by Popescu et al., which is also based on the U-Net network for predicting brain age. Secondly, to assess the impact of using different brain regions as input features on age prediction, separate training was conducted using the entire brain and two specific brain regions (the hippocampus and amygdala) with a 3D ResNet 34 model. Furthermore, the hippocampus and amygdala were combined and input into the multi-channel input network model for comparative experiments. The selected brain regions were chosen based on medical prior knowledge, and numerous studies suggest a high correlation between these two brain regions and aging. Additionally, we used the ADNI datasets to explore the changes in personalized brain regions in response to disease progression. We employed a random sampling approach to partition 80% of the Cam-CAN dataset as the training set (denoted as NC-train), while allocating the remaining 20% as the validation set (labeled as NC-val) for model training. Subsequently, the trained model was applied to two independent test sets, denoted as MCI-test and AD-test, each comprising 56 individuals diagnosed with MCI and AD, respectively. The losses on three evaluation metrics (MAE, RMSE, MSE) for these two independent test sets were computed and subjected to comprehensive analysis and discussion. Finally, in order to examine the correlation between the chosen brain regions and the severity of brain aging, we employ the ADNI dataset. Following the segmentation of samples, we acquire data pertaining to the hippocampus and amygdala. Subsequently, we conduct age prediction for both the hippocampus and amygdala, compute the MAE, and generate heatmaps.

The experiments were run on a server with a single Tesla V100-SXM2-32GB GPU, using CUDA version 10.3 and Python as the programming language. The model was implemented using the PyTorch 1.3.0 framework. The Adam optimizer was employed with a learning rate of 0.001, the number of epochs set to 60, and a batch size of 4.

## Result and discussion

### Comparison with other models

First, cropped images with dimensions of 128 × 128 × 128 were used as input data for Tri-UNet and two baseline models (U-Net and ResNet 34). Tri-UNet achieved a minimum Mean Absolute Error (MAE) of 7.46 years. Compared to the best MAE of the brain age prediction network proposed by Popescu et al.^22^. (9.5 years), Tri-UNet showed an improvement of 2.04 years. Subsequently, cropped images with dimensions of 32 × 32 × 32 were used as input data for Tri-UNet and the two baseline models, conducting ablation experiments. Finally, a comparative experiment between single-channel input networks and multi-channel input networks was performed using ResNet34.

The experimental results for input data with dimensions of 128 × 128 × 128 are shown in Table 2. Tri-UNetResNet denotes the model where ResNet34 is concatenated directly after the Tri-UNet network for brain age prediction. The results demonstrate the effectiveness of the proposed Tri-UNet method in the task of predicting brain age.

**Table 2:** Comparison of Tri-UNet with other models.

Models	Dataset	MinMAE	MaxMAE	MeanMAE
U-Net	Whole Brain	15.05	43.21	17.23
ResNet34	Whole Brain	9.17	36.6	12.42
Tri-UNet	Whole Brain	**7.46**	**16.9**	**10.05**

Significant values are in bold.

When the input data size is 128 × 128 × 128, as the results predicted by U-Net are far less favorable compared to ResNet34 and the proposed model Tri-UNet concatenated with ResNet34, the comparison is made only between ResNet34 and the proposed model in other evaluation metrics. The experimental results are shown in Table 3.

**Table 3:** Comparison between Tri-UNet and ResNet34.

Models	Dataset	MAE	RMSE	MSE
ResNet34	Whole brain	9.17	11.47	131.68
Tri-UNet	Whole brain	**7.46**	**9.32**	**86.96**

Significant values are in bold.

As depicted in Table 3, the Tri-UNet model demonstrates superior performance over ResNet34 in forecasting brain-related data, exhibiting lower MAE, RMSE, and MSE scores.

### Result of single-channel input network

To validate the performance of the multi-channel network and the approach using brain region segmentation maps obtained with medical prior knowledge as input data, a comparative experiment was conducted on the 3D ResNet 34 model using single-channel input. The input data consisted of untrimmed images (size: 256 × 256 × 256), and the experimental results are shown in Table 4.

**Table 4:** Results of the single-channel input network (256 × 256 × 256).

Dataset	MinMAE	MaxMAE	MeanMAE
Whole brain	**4.98**	**115.42**	22.4
AM	8.26	225.95	24.91
HA	6.95	154.43	18.78
HBT	9.07	160.46	**18.02**

Significant values are in bold.

As depicted in Table 4, the HBT dataset exhibits the lowest MeanMAE, standing at a mere 18.02, whereas the AM dataset records the highest MeanMAE.

When the input data size is 32 × 32 × 32, similarly, the performance of the single-channel network was validated on the 3D ResNet 34 model, obtaining different evaluation metrics. The experimental results are shown in Table 5.

**Table 5:** Results of the single-channel input network (32 × 32 × 32).

Dataset	MAE	RMSE	MSE
Whole brain	**15.18**	**17.56**	**308.54**
HA	16.7	19.18	367.99

Significant values are in bold.

As depicted in Table 5, HA's MAE, RMSE, and MSE are all higher than those of the Whole Brain, at 16.7, 19.18, and 367.99 respectively. This indicates that HA's prediction errors are greater than those of the Whole Brain. Specifically, HA's MAE, RMSE, and MSE are all relatively large, suggesting that the prediction model for HA may have poor performance and requires further optimization.

To validate the performance of the multi-channel input network and the method of using anatomically informed brain region segmentation maps as input data, a comparative experiment was conducted on the 3D ResNet 34 model. The entire dataset used untrimmed images (size: 256 × 256 × 256), and the experimental results are presented in Table 6.

**Table 6:** Results of the multi-channel input network.

Dataset	MinMAE	MaxMAE	MeanMAE
HA L + R	8.6	285.96	30.54
HA + AM	**8.6**	**165.65**	**22.12**
HA + Whole brain	14.47	224.45	31.66

Significant values are in bold.

As depicted in Table 6, the HA + AM dataset has the lowest MeanMAE, at 22.12, while the HA + Whole Brain dataset has the highest MeanMAE, at 31.66. This means that the average prediction error of the HA + AM dataset is the smallest, while that of the HA + Whole Brain dataset is the largest.

### Result of different input

**Figure 6:**
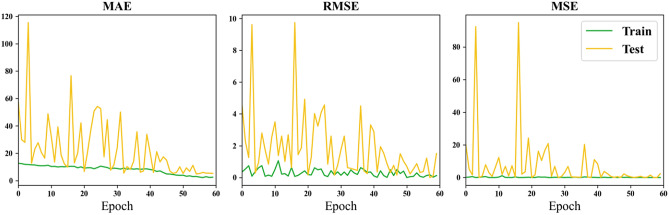
Loss curve of using the whole brain as input.

Figure 6 illustrates the changes in MAE, RMSE, and MSE loss metrics over training epochs when using the entire brain as input data.

**Figure 7:**
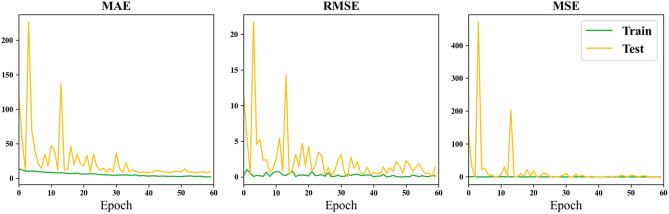
Loss curve of using the amygdala as input.

Figure 7 illustrates the evolution of MAE, RMSE, and MSE metrics with the progression of training epochs, utilizing the Amygdala (AM) as input data.

**Figure 8:**
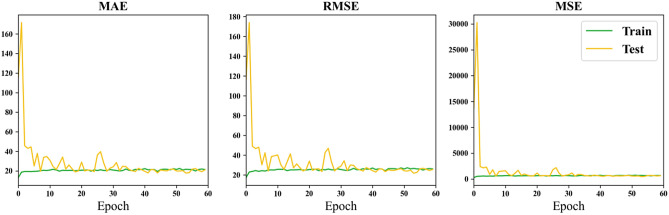
Loss curve of using the hippocampus as input.

Figure 8 depicts the variations in MAE, RMSE, and MSE metrics with the increase in training epochs, employing the Hippocampus (HA) as input data.

Through the comparison of results for the whole brain, hippocampus (HA), and amygdala presented in Table 4, it is observed that the minimum MAE for the whole brain is 4.98 years, reaching the lowest value among all experimental outcomes. In contrast, the minimum MAE for HA is 6.95 years, and for AM, it is 8.26 years. This difference may be attributed to the information richness provided by the whole brain as input data, potentially yielding more accurate results in certain rounds of training. Deep learning, which involves the neural network learning detailed features, benefits from a larger Region of Interest (ROI) to enhance global feature representation. The larger ROI or whole brain as input features leads to a larger receptive field, resulting in better predictive performance for brain age. Furthermore, from Table 4, it is observed that the minimum MAE for HA is lower than that for AM, suggesting that HA is more correlated with age compared to AM. However, in terms of average MAE, the whole brain has an average MAE of 22.40, HA has an average MAE of 18.78, and AM has an average MAE of 24.91. This indicates that larger regions may offer more information for more accurate predictions, but sometimes the smaller region chosen brings less noise. Therefore, a balance must be struck between obtaining more information and achieving finer segmentation features of brain regions. Comparing the curves in Figs. 5, 6, and 7, it can be observed that both HA and AM provide less information than the whole brain, resulting in faster convergence of the models, confirming the above conclusions.

**Figure 9:**
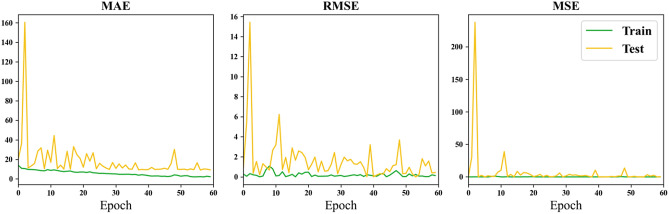
Loss curve of using high-resolution hippocampus as input.

Additionally, this study used a high-resolution method to segment the hippocampus (HBT) and used it as input data, as shown in Fig. 9 displaying the loss change curve. From the figure, it is evident that the minimum MAE results for predicting brain age using high-resolution hippocampal images as input data are not as good as those obtained with low-resolution hippocampus (HA) as input data. The minimum MAE for HBT is 9.07 years, which is 2.12 years higher than the result for HA. This may be attributed to the richer information content in high-resolution images, which also introduces more redundant information. This finding aligns with the results of the comparison experiment between the whole brain and HA, providing mutual confirmation for the observed reasons.

**Figure 10:**
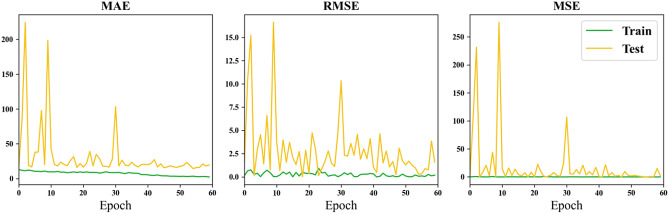
Loss curve of using both the whole brain and hippocampus as input.

Hence, to confirm this hypothesis, the whole brain and hippocampus (HA) were used together as input for the multi-channel network to predict the brain age. The resulting loss change curve is shown in Fig. 10.

Observing the case where the whole brain and hippocampus are used together as input, the minimum MAE is 14.47 years. The result is neither as good as training the model with the whole brain alone (MAE of 4.98 years) nor as good as training the model with the hippocampus alone (MAE of 6.95 years). This validates the hypothesis in this study that in the task of predicting brain age, finer voxel predictions do not necessarily yield better results, and including more features does not guarantee better outcomes. In the presence of redundant features, the results may not be as good as those obtained with individual features.

**Figure 11:**
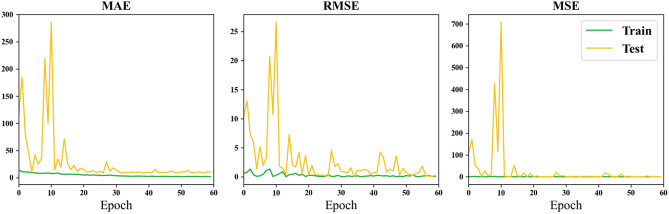
Loss curve of using the left and right parts of the hippocampus separately as input.

Therefore, separating the left and right sides of the hippocampus (HA L + R) as input for the multi-channel network, the loss change curves for the three evaluation metrics (MAE, RMSE, MSE) with increasing training epochs are shown in Fig. 11. Combining the results in Table 6, it can be observed that separating the left and right sides of the hippocampus (HA L + R) as input for the dual-channel network does not perform as well as inputting the hippocampus alone. This may be due to both providing the same amount of information, but separating the sides increases the regions with pixel intensity values of 0, resulting in more noise and, therefore, less effective results compared to inputting the hippocampus alone. Trimming off the regions with intensity values of 0 might improve the performance.

**Figure 12:**
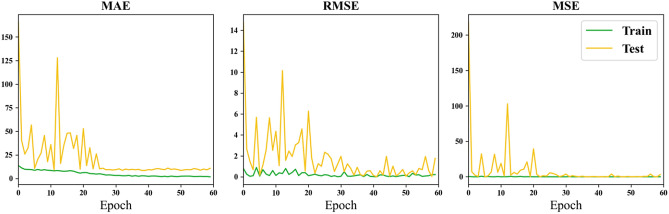
Loss curve of using the hippocampus and amygdala together as input.

Finally, using the hippocampus and amygdala (HA + AM) as input for the multi-channel network, the loss change curves for the three evaluation metrics (MAE, RMSE, MSE) with increasing training epochs are shown in Fig. 12.

**Figure 13:**
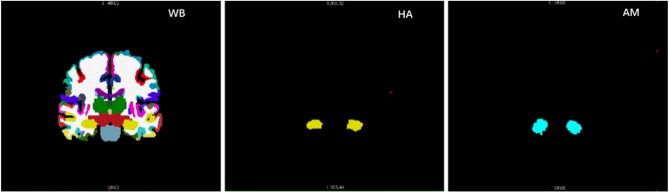
Comparison of the whole brain with the hippocampus and amygdala anatomical structures.

Combining the information from Table 6, it is observed that the minimum MAE when using the hippocampus and amygdala together as input for the multi-channel network is 8.60 years. This is slightly higher than the minimum MAE with the entire brain as input (4.98 years). However, the average MAE improves to 22.12 years compared to 22.40 years for the entire brain, demonstrating that a multi-channel input network can enhance stability in performance by combining different data. Nevertheless, as the input images are of the same size, the double-channel input images contain many more regions with pixel grayscale values of 0, as shown in Fig. 13. In the future, it would be beneficial to crop out the informative regions as input, which may lead to better results.

### Result on degenerative diseases datasets

Alzheimer's disease (AD) is a typical neurodegenerative disorder characterized by cognitive impairment accompanied by memory loss, language difficulties, and decline in neurological function. In this study, the task of predicting brain age was performed on three datasets HC, MCI and AD. Two datasets consisting of healthy subjects were used to train and validate the model, and then tested on two independent test sets MCI and AD. The losses on three evaluation metrics for both patient groups were obtained to assess the degree of brain aging in diseased individuals, thereby validating the effectiveness of brain age prediction as an auxiliary tool for clinical diagnosis of mild cognitive impairment (MCI) and Alzheimer's disease by clinicians. The experimental results are presented in Table 7.

**Table 7:** Result on ADNI dataset.

Dataset	MAE	RMSE	MSE
MCI-test	6.26	7.88	61.98
AD-test	**7.27**	**9.08**	**82.52**

Significant values are in bold.

**Figure 14:**
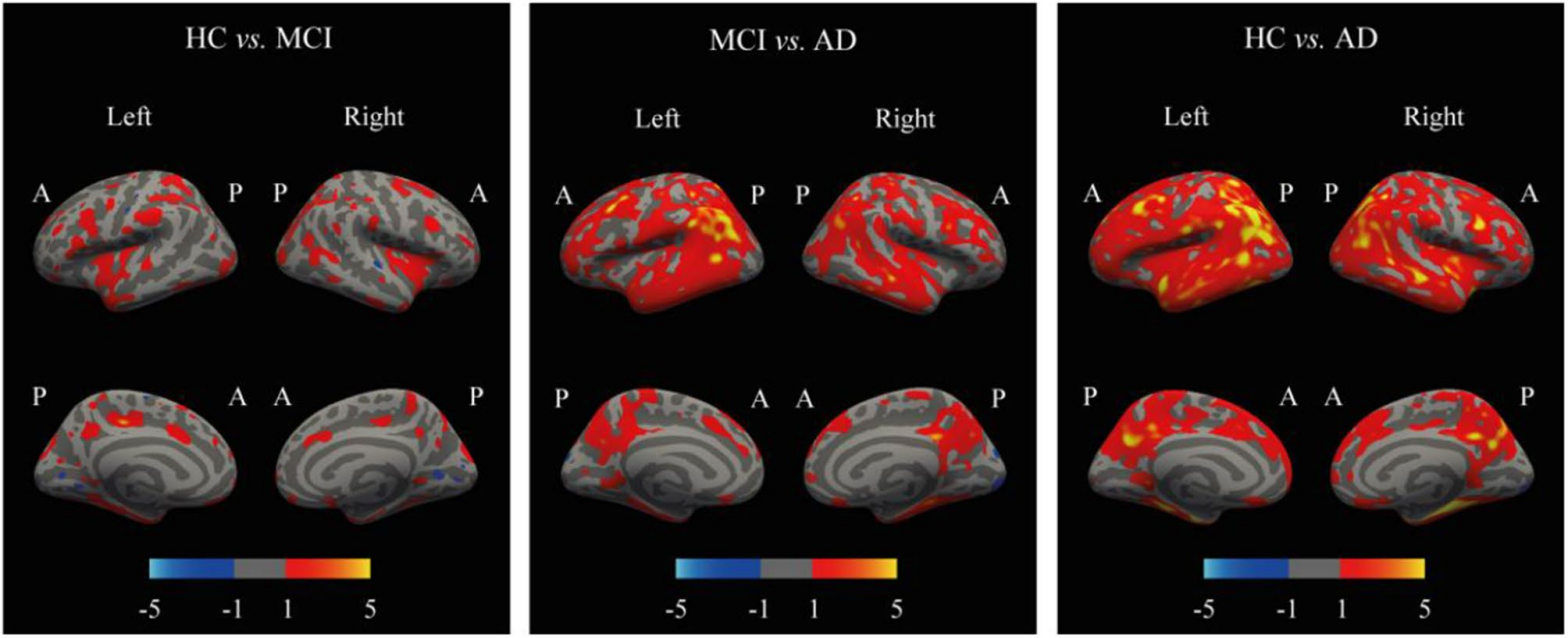
Three groups of subjects' pairwise analysis heatmap.

The results of the model on the two independent test sets are shown in Table 7. It can be observed that the results for the AD group on all three evaluation metrics are greater than those of the MCI group, indicating that the degree of brain atrophy in the AD group is greater than that of the MCI group participants, consistent with findings in other relevant studies. The experimental results demonstrate that in the task of predicting brain age, the difference between the predicted age and the actual age of participants with Alzheimer's disease is greater than that of participants with mild cognitive impairment, indicating a greater degree of brain aging in participants with Alzheimer's disease compared to those with mild cognitive impairment. Additionally, through the heatmap analysis of pairwise comparisons between HC and MCI, MCI and AD, HC and AD groups (as shown in Fig. 14), it is further demonstrated that the degree of brain atrophy in MCI patients is greater than that in HC participants, and the degree of brain atrophy in AD patients is greater than that in both MCI patients and HC participants.

**Figure 15:**
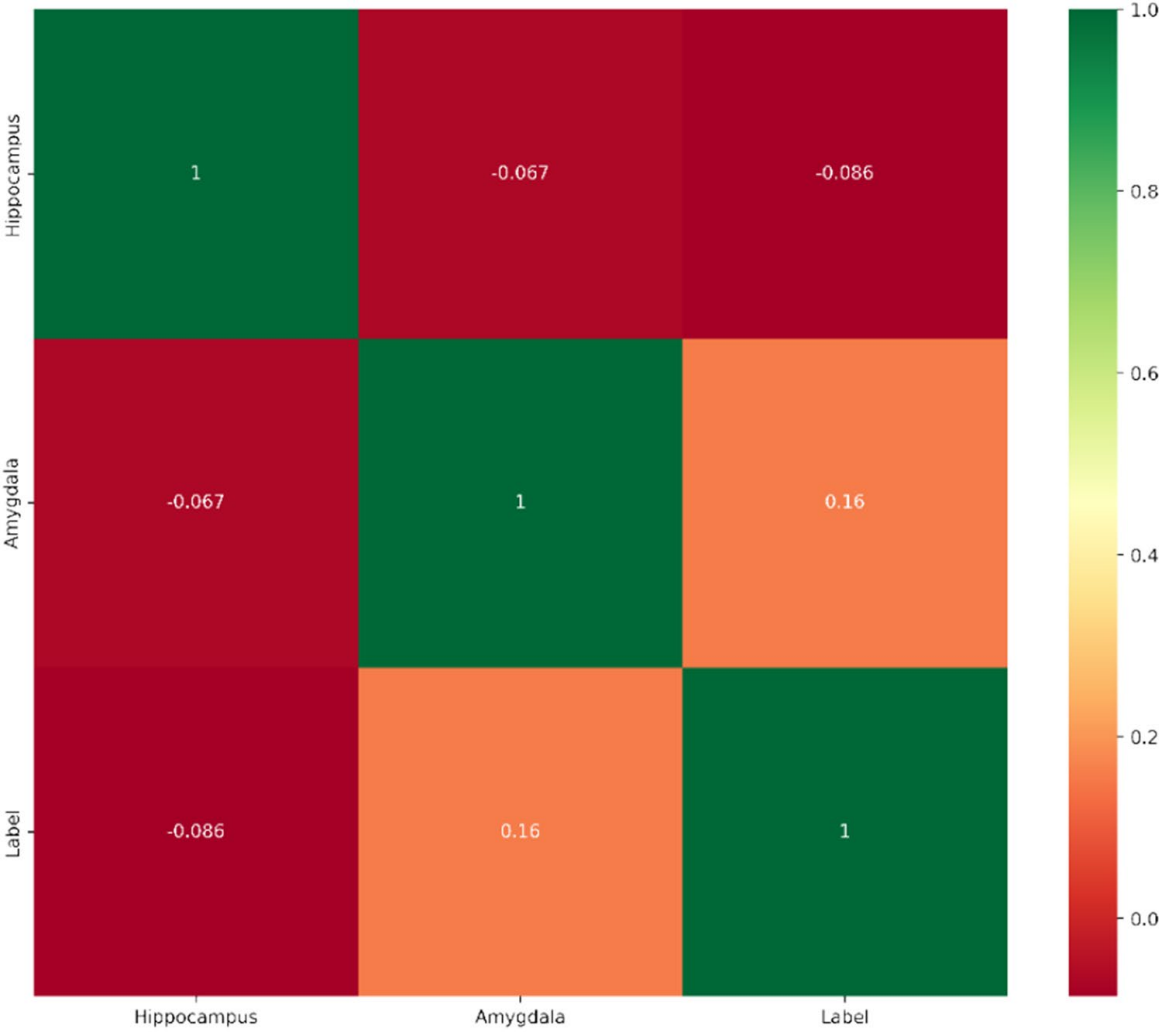
Heatmap of different brain age with the severity of brain aging abnormalities.

To explore the correlation between different brain regions and the severity of brain aging abnormalities, we plotted a heatmap of the hippocampus, amygdala, and brain disease status. As shown in Fig. 15, the correlation coefficient between Hippocampus and Label is -0.086, indicating a negative correlation, albeit very weak. This may suggest a slight negative correlation between hippocampal measurements and the degree of brain aging. Finally, there is a positive correlation of 0.16 between Amygdala and Label. This suggests that as the age of the amygdala increases, there may be an increase in the severity of brain aging abnormalities.

## Conclusion

This chapter introduces a brain age prediction model, Tri-UNet, based on 3D U-Net and 3D ResNet. The model incorporates residual connections from ResNet, reusing features multiple times on the basis of U-Net, enhancing the correlation between features at different scales, and achieving optimal feature utilization, resulting in good overall performance. Additionally, a multi-channel input network model based on 3D ResNet is proposed, predicting brain age by inputting 3D brain region data determined by anatomical prior knowledge. This approach addresses issues in existing work related to information redundancy or segmentation regions lacking anatomical principles. Experimental results demonstrate an improved performance of the proposed brain age prediction framework compared to existing methods, positioning it as a valuable auxiliary tool in clinical medical research. Despite these advantages, Due to variations in the degree of atrophy and its correlation with age across different brain regions, Tri-UNet cannot make more accurate predictions. Therefore, in our subsequent work, we will enhance Tri-UNet's ability to determine the brain regions to which voxel blocks belong, and combine this with assigning different weights to brain regions for prediction purposes.

## Data Availability

The datasets generated and/or analyzed during the current study are available in the Cambridge Centre for Ageing and Neuroscience (Cam-CAN) data repository (https://www.cam-can.com/index.php?content=dataset) and Alzheimer’s Disease Neuroimaging Initiative (ADNI) data repository (http://adni.loni.usc.edu/about/).
